# Prophylactic “First-Step” Central Neck Dissection (Level 6) Does Not Increase Morbidity After (Total) Thyroidectomy

**DOI:** 10.1245/s10434-016-5338-5

**Published:** 2016-07-08

**Authors:** Andreas Selberherr, Philipp Riss, Christian Scheuba, Bruno Niederle

**Affiliations:** Section of Endocrine Surgery, Division of General Surgery, Department of Surgery, Medical University, Vienna, Austria

## Abstract

**Background:**

In terms of morbidity, prophylactic central neck dissection (CND; level 6) in potentially malignant thyroid disease is discussed controversially. The rates of (transient and permanent) hypoparathyroidism and palsy of the recurrent laryngeal nerve (RLN) after “first-step” (FS-)CND are analyzed in this study.

**Methods:**

Bilateral and unilateral FSCND, i.e., lymph node dissection along the RLN before (total) thyroidectomy, was performed bilaterally in 68 (group 1) and unilaterally in 44 patients (group 2), respectively. The rates of hypoparathyroidism and palsy of the RLN were documented prospectively and were compared to 237 patients of group 3 (controls) who underwent (total) thyroidectomy only.

**Results:**

Fifteen of 68 patients (22 %) of group 1 developed transient and one patient had permanent hypoparathyroidism. Transient unilateral palsy of the RLN was observed in ten patients (15 %); none were permanent. Transient hypoparathyroidism was monitored in 10 of 44 patients (23 %) of group 2 and permanent hypoparathyroidism in 1 (2 %). Six patients (14 %) developed temporary palsy of the RLN; one remained permanent. Palsy was seen in 3 patients on the contralateral side of unilateral FSCND. Transient and permanent hypoparathyroidism was observed in 50 (21 %) and 2 (1 %) of 237 controls. Transient palsy of the RLN was documented in 22 (9 %) of 237 controls and permanent palsy of the RLN in 4 (2 %).

**Conclusions:**

In this single-center series, the overall permanent morbidity was low (1 %). Therefore, FSCND may be recommended (even prophylactically) for experienced high-volume surgeons in patients with thyroid nodules suspicious for malignancy.

Surgery for thyroid cancer includes lymph node dissection of the central neck compartment (CND; level 6). This involves prelaryngeal, pretracheal, as well as lymph nodes along and around both recurrent laryngeal nerves (RLN) after removing the thyroid gland.^1^ Reviewing the literature, many reports have summarized arguments for and against this procedure in (suspected) follicular-derived thyroid cancer.^2^ It is a wide consensus that therapeutic initial CND is meaningful in cytologically proven malignant diseases, as unsuspected metastatic lesions in the central neck compartment are seen in up to 40 % of cases.^3^–^5^ Prophylactic/therapeutic CND is recommended to avoid the complications of reoperation and to establish adequate staging to indicate further therapeutic options (i.e., radioiodine ablation).^6^ In medullary thyroid carcinoma (MTC), the prognostic value of (prophylactic) CND has been documented.^7^

The purpose of this prospective observational study was to evaluate the rate of permanent hypoparathyroidism and palsy of the RLN in patients after (total) thyroidectomy (TT) and “first-step” (FS-) unilateral and bilateral CND compared to complications in patients with TT. The controversies regarding whether to perform or not to perform prophylactic CND are not discussed.

## Patients and Methods

A systematic analysis of a prospectively maintained database comprising 112 patients who had received initial TT and bilateral (group 1) or unilateral (group 2) FSCND was performed and compared to a “control group” of 237 patients who had received TT only (group 3). The patients were treated during a 3-year study period.

### Patients

#### Thyroid Metabolism

All patients had normal thyroid-stimulating hormone, Free T3, and Free T4 values.

#### Preoperative Ultrasonography

Preoperative ultrasonography of the thyroid gland and the central lymph node compartment (level 6) was performed in all patients. Bilateral thyroid nodules were documented in all patients. There was no evidence of enlarged and thus suspicious lymph nodes in the central neck (clinically/radiologically N0 [c/r N0]).

Group 1: Bilateral FSCND was performed in 68 patients (40 females, 28 males; mean age 42 years [range 9–81]) with biochemically verified or suspected MTC, as well as patients with papillary thyroid carcinoma (PTC) diagnosed by fine-needle aspiration biopsy and classified as Bethesda VI (malignant).^8^

Group 2: Unilateral FSCND was performed in 44 patients (34 females, 10 males; mean age 52 years [range 15–87]) with thyroid nodules classified as “indeterminate tumors” by fine-needle aspiration biopsy (Bethesda III–V). In groups 1 and 2, a mean of 9 (range: 1–33) lymph nodes were removed during uni- or bilateral FSCND.

Group 3: A total of 237 patients (191 females, 46 males; mean age 54 years [range 16–87]) with benign bilateral multinodular goiter and TT without FSCND provided as controls. Concerning average gland weight and severity of thyroiditis, there were no significant differences between the three groups.

### Surgical Technique

#### First-Step Central Neck Dissection (FSCND)

By definition, lymphadenectomy of prelaryngeal, pretracheal, and unilateral or bilateral along the RLN (CND; clearance of level 6) was performed after extended mobilization of the upper and lower poles of the thyroid gland.^9^ Pulling the thyroid lobe upward and medially allowed optimal palpation, visualization, and dissection of the RLN. Before removing the thyroid lobe, the lymphatic tissue was dissected along the RLN. This surgical maneuver was performed prophylactically before removing one or both thyroid lobes.

The surgical procedures were performed in a university hospital (the Section of Endocrine Surgery, a tertiary referral center) by two experienced endocrine surgeons (C.S. and B.N.; case load/year: >100 procedures). All patients gave informed consent to all diagnostic and therapeutic procedures. The prospective data collection and the retrospective analysis were approved by the Ethics Committee of the Medical University of Vienna (approval number 1943/2012).

### Parathyroid Autotransplantation

Whenever feasible, all four parathyroid glands were visualized and carefully dissected from the thyroid capsule. Once the blood supply was preserved, the parathyroids were kept in situ. If the blood supply of a parathyroid gland could not be maintained or hypovascularization was suspected, the gland was removed and organ diagnosis was confirmed by frozen section. The gland was subsequently stored at 4 °C in 0.9 % NaCl until the end of the surgical procedure and then immediately autotransplanted into the sternocleidomastoid muscle according to a standard procedure before closing the wound.^10^

In all patients with parathyroid autotransplantation, at least two additional parathyroids were visualized during surgery and left in situ with an optimal vascular supply. Synchronous parathyroid autotransplantation was performed in 30 of 68 patients (44 %) of group 1, in 9 of 44 patients (20 %) of group 2, and in 53 of 237 patients (22 %) of group 3. The rate of parathyroid autotransplantation was significantly higher in the group with bilateral FSCND (*p* ≤ 0.05).

### Frozen Section

Frozen section was performed in all specimens. Only patients with “initial FSCND” were included in the analysis. If malignancy was proven intraoperatively, CND was extended to the contralateral side as a “second step procedure.” These patients were excluded from this comparative analysis.

### Parathyroid Metabolism

Measurement of albumin-adjusted total serum calcium (sCa), intact parathyroid hormone (iPTH), and creatinine was performed preoperatively, in the morning of the first postoperative day, and during follow-up 1 and 6 months postoperatively.^11^ SCa (normal range: 2.0–2.6 mmol/l) was measured on a conventional standard autoanalyzer and iPTH (normal range: 15–65 pg/ml) using a commercially available iPTH assay (Elecsys 1010; Roche Diagnostics; Mannheim, Germany).

### Follow-up

Patients with a disturbed parathyroid metabolism on the first postoperative day and/or a limited function of the recurrent laryngeal nerve between days 1 and 5 postoperatively were followed at least 1 year (see definitions below).

### Definition of Postoperative Hypoparathyroidism

Postoperative hypoparathyroidism was defined as parathyroid hormone levels <15 pg/ml on the first postoperative day with coincident inadequately low calcium and the need for calcium and vitamin D supplementation regardless of the presence of symptoms.^11^ Postoperative hypoparathyroidism was defined as “transient” if iPTH and sCa normalized within 6 months. When serum levels of iPTH and Ca were still below the reference range and if patients were taking calcium and vitamin D medication after 6 months, hypoparathyroidism was defined as “permanent.”

### Laryngological Evaluation

FSCND was accompanied by extended dissection of the (inferior) RLN without intraoperative neuromonitoring.^12^ All patients presented with a normal preoperative level of vocal cord mobility. Between days 1 and 5, postoperative laryngoscopy was performed routinely.

### Definition of a Postoperative Disturbance of the RLN Function

Irritation of the RLN was classified as transient in the presence of temporary hoarseness based on vocal cord immobility at laryngoscopy. When vocal cord immobility was observed, further follow-up to classify transient/permanent RLN palsy was applied. Permanent hoarseness accompanied by paralysis of the vocal cord(s) at laryngoscopy for longer than 6 months was defined as a permanent complication.

### Statistical Analysis

Data were analyzed by descriptive statistics and the Chi square test using Microsoft Excel (Microsoft Corporation, Roselle, IL). The level of significance was set at *p* < 0.05.

## Results

### Morbidity

Hypoparathyroidism and paralysis of the RLN in the three patient groups are summarized in Table 1.

**Table 1:** Overview of complication rates depicting insignificant differences between the groups

	n	Hypoparathyroidism n (%)		Recurrent laryngeal nerve palsy n (%)	
		Transient	Permanent	Transient	Permanent
Group 1 (thyroidectomy + bilateral FSCND)	68	15 (22)	1 (1)	10 (15)	0
Group 2 (thyroidectomy + unilateral FSCND)	44	10 (23)	1 (2)	6 (14)	1 (2)
Group 3 (thyroidectomy)	237	50 (21)	2 (1)	22 (9)	4 (2)
Total	349	75 (21)	4 (1)	38 (11)	5 (1)

*FSCND* first-step central neck dissection

### Postoperative Hypoparathyroidism and Parathyroid Autotransplantation

Synchronous parathyroid autotransplantation was performed in 30 of 68 patients (44 %) of group 1, in 9 of 44 patients (20 %) of group 2, and in 53 of 237 patients (22 %) of group 3. The rate of parathyroid autotransplantation was significantly higher in the group with bilateral FSCND (*p* < 0.05).

Fifteen patients (22 %) of group 1 developed transient and one (1 %) permanent hypoparathyroidism. In group 2, ten patients (23 %) developed transient and one (2 %) permanent hypoparathyroidism. In group 3, temporary and permanent hypoparathyroidism were seen in 50 (21 %) and 2 (1 %) patients, respectively. Comparing groups 1 and 2 with group 3, no significant differences were identified.

### Palsy of the RLN

Unilateral transient palsy of the RLN was observed in ten patients (15 %) of group 1. Neither bilateral nor permanent paresis was observed in this patients group.

In group 2, six patients (14 %) developed transient palsy of the RLN, one (2 %) remained permanent. The nerve palsy was documented in three of the six patients on the side of FSCND (including the permanent palsy). In the other three patients, the transient nerve palsy was described on the contralateral side of the FSCND.

In group 3, 22 of 237 patients (9 %) developed transient and 4 (2 %) permanent unilateral palsy of the RLN.

Details concerning transient and permanent palsy of the RLN subdividing patients with nerves at “high risk” (FSCND) and patients with nerves at “normal risk” (no FSCND) are depicted in Table 2. No statistical significance was documented comparing groups 1 and 2 with group 3.

**Table 2:** Subdivision of patients with nerves at “high risk” (FSCND) and patients with nerves at “normal risk” (no FSCND)

Group	Patients	Nerves at risk	Nerves at high/normal risk				Nerves at high risk (=with FSCND)				Nerves at normal risk (=without FSCND)			
	n	n	Transient		Permanent		Transient		Permanent		Transient		Permanent	
			n	%	n	%	n	%	n	%	n	%	n	%
I	68	136	10/136	7.4	0	0	10/136	7.4	0	0				
II	44	88	6/88	6.8	1/88	1.1	3/44	6.8	1/44	2.3	3/44	6.8	0	0
III	237	474	22/474	4.8	4/474	0.8					22/474	4.8	4/474	0.8
Σ	349	698	38/698	5.4	5/698	0.7	13/180	7.2	1/180	0.6	27/518	5.2	4/518	0.7

*FSCND* first-step central neck dissection

### Final Histological Results

As summarized in Table 3 (not including patients with TT without CND and therefore unknown lymph nodes status), the final histological reports revealed various types of malignancy in 55 of 68 patients (81 %) of group 1 and in 25 of 44 patients (57 %) of group 2, respectively. Overall, lymph-node metastases (mean 5; range 1–16 nodes) were documented in 39 of 80 patients (49 %), in 35 (51 %) of group 1, and in 4 (9 %) of group 2, respectively.

**Table 3:** Final histologic evaluations (not including patients with TT without FSCND)

Surgical procedure	Final histological results			
	N	Carcinoma with lymph node metastasis	Carcinoma without lymph node involvement	Benign
Thyroidectomy + bilateral FSCND	68	35 (51 %)	20 (29 %)	13 (19 %)
Thyroidectomy + unilateral FSCND	44	4 (9 %)	21 (48 %)	19 (43 %)

*FSCND* first-step central neck dissection

## Discussion

Many surgeons avoid (prophylactic) CND as an initial procedure in suspected or verified malignant thyroid micro-disease (tumor diameter ≤10 mm; UICC 2009 pT1a) due to an overall high complication rate, although a high rate of clinically and sonographically unsuspected lymph node metastasis may be revealed.^13^–^15^ However, the majority of authors have recommended (prophylactic/therapeutic) CND in malignant thyroid macro-disease (tumor diameter >11 mm; UICC 2009 ≥pT1b).^16^

In this single-center study, uni- or bilateral CND as a first-step procedure was performed prophylactically in nodules with a “high risk of malignancy” and c/r N0.^9^ The morbidity associated with extended prophylactic CND was compared to the lesser extent of applied lymph node surgery. The overall rate of permanent morbidity was 1 %, with no significant difference between patients with or without CND.

Following TT, *transient and permanent hypoparathyroidism* were documented in 21 and 1 % of cases, respectively. There was no statistically significant difference between procedures without (21 and 1 %) and with unilateral (23 and 2 %) and/or bilateral FSCND (22 and 1 %). A standardized protocol was applied in the current study to diagnose and define temporary and permanent postoperative hypoparathyroidism based on PTH levels after TT.^11^

Depending on the definition based on calcium alone or on calcium in combination with PTH, there have been variations in reporting temporary and permanent hypoparathyroidism in various studies, thus making it difficult to compare the complication rates. In the literature, however, transient hypoparathyroidism has been reported to be between 9.7 and 56.5 % after TT with bilateral CND (Table 4). The same authors have described this complication in 4–33.6 % of cases, necessitating temporary calcium and vitamin D substitution after simple TT. A permanent disturbance of the parathyroid metabolism has been reported in up to 19.4 % after CND compared to between 0.6 and 8.1 % after simple TT.

**Table 4:** Complications according to nerves/parathyroid sides at risk

Author	Year	Hypoparathyroidism				Paralysis recurrent nerve				N positive
		Transient		Permanent		Transient		Permanent		
		TT	TT + CND	TT	TT + CND	TT	TT + CND	TT	TT + CND	
		n (%)	n (%)	n (%)	n(%)	n (%)	n (%)	n (%)	n (%)	n patients(%)
Selberherr (this study)	2016	50/237 (21.1)^11^	25/180 (13.9)	2/237 (0.8)^11^	2/180 (1.1)	22/237 (9.3)	16/180 (8.9)	4/237 (1.7)	1/180 (0.6)	39/80 (48.8)
Zhang^15^	2015	10/108 (9.3)	40/134 (29.9)	0	2/134 (1.5)	1/108 (0.9)	2/134 (1.5)	1/108 (0.9)	1/134 (0.7)	51/134 (38.1)
Viola^29^	2015	n.a.	n.a.	7/88 (8.0)	18/93 (19.4)	n.a.	n.a.	7/88 (8.0)	4/93 (4.3)	43/93 (46.2)
Barczynski^22^	2013	39/282 (13.8)	117/358 (32.7)	2/282 (0.7)	8/358 (2.2)	24/282 (8.5)	35/358 (9.8)	6/282 (2.1)	9/358 (2.5)	108/640 (16.9)
Laird^30^	2012	n.a.	?/118	n.a.	2/118 (1.7)	n.a.	2/118 (1.7)	n.a.	0 (1**)/118	67/118 (56.8)
Lang^37^	2012	9/103 (8.7)	15/82 (18.3)	1/103 (1.0)	2/82 (2.4)	0/103	3/82 (3.7)	1/103 (1.0)	1/82 (1.2)	45/82 (54.9)
Raffaelli^31^	2012	11/62 (17.7)	35/62 (56.5)	0/62	1/62 (1.6)	0/62	0/62	0/62	0/62	26/62 (41.9)
So^32^	2012	38/113 (33.6)	49/119 (41.2)	2/113 (1.8)	7/119 (5.9)	4/113 (3.5)	4/119 (3.4)	2/113 (1.8)	1/119 (0.8)	44/119 (37.0)
Giordan 23	2012	109/394 (27.7)	160/308 (51.9)	25/394 (6.3)	50/308 (16.2)	14/394 (3.6)	17/308 (5.5)	4/394 (1.0)	7/308 (2.3)	n.a.
Wang^5^	2012	4/37 (10.8)	21/49 (42.9)	3/37 (8.1)	0/49	n.a.	n.a.	1/37 (2.7)	1/49 (2.0)	20/49 (40.8)
Roh^33^	2011	n.a.	49/184 (26.6)	n.a.	3/184 (1.6)	n.a.	2(6*)/184 (1.1)	n.a.	0 (4*)/184	79 (80)/(167) 184 (min. 42.9 %)
Popadich^34^	2011	14/347 (4.0)	25/259 (9.7)	2/347 (0.6)	2/259 (0.8)	8/347 (2.3)	1/259 (0.4)	6/347 (1.7)	1/259 (0.4)	127/259 (49.0 %)

*TT* (total) thyroidectomy, *CND* central neck dissection

* Nerve intentionally resected in four patients

** Nerve intentionally resected

In a recently published meta-analysis of prophylactic CND, temporary hypocalcemia was the major surgical morbidity in patients with prophylactic CND, yet when excluded, overall morbidity appeared similar comparing the patients groups who had undergone TT with CND.^17^

Every effort is to be made to preserve viable parathyroid tissue, in particular the superior glands, as the inferior glands are more likely to be damaged or excised in the course of CND. Prophylactic parathyroid autotransplantation may reduce permanent hypoparathyroidism.^18^,^19^

In the current analysis, a significant difference was identified between the groups regarding the rates of parathyroid autotransplantation. As expected, the number of autotransplanted patients was significantly higher in the presence of bilateral FSCND, indicating that parathyroid autotransplantation of one gland may neither decrease nor increase the rate of transient or permanent hypoparathyroidism if at least two additional parathyroids are left in situ and well vascularized.^20^

* Transient and permanent unilateral paralysis* of the RLN nerve were revealed in 8.9 and 0.6 % of nerves at risk, respectively. There was no statistical significance comparing procedures with and without FSCND.

Data in the literature have reported temporary or permanent paralysis of the RLN after simple TT of up to 8 % and, in more extended procedures, of between 0.4 and 9.8 %. The addition of CND to TT has been reported to increase neither the risk of temporary nor of permanent vocal cord paralysis in prophylactic or therapeutic procedures.^17^,^21^–^37^

Because of the inhomogeneous patients groups described in the literature and the lack of routine postoperative laryngoscopy in many studies, no statistical comparison with the current analysis was possible. However, primary (uni- or bilateral) FSCND comprises—in accordance with literature—at least the same or even a lower rate of permanent paralysis.

In MTC, CND is recommended in various international guidelines but is still a subject of controversy in PTC management regarding routine or therapeutic indications and its impact on local recurrence and survival. The overall low rate of permanent morbidity in this study warrants the recommendation of (even prophylactic) FSCND in all patients with suspicious thyroid nodules, thus improving cancer staging and avoiding more complicated second step reoperations comprising a higher level of (permanent) complications. However, these data represent the unique experience of a specialized high-volume centre and may not be transferable to other institutions with lower caseloads.
